# Giant rapidly involuting congenital hemangioma in a neonate: a case report

**DOI:** 10.3389/fped.2026.1780760

**Published:** 2026-05-29

**Authors:** Jian Pang, Yulan Pang, Yanni Tang, Pingping Liu, Shuihua Yang

**Affiliations:** Department of Medical Ultrasonics, Maternal and Child Health Care Hospital of Guangxi Zhuang Autonomous Region, Nanning, China

**Keywords:** conservative management, giant, lower extremity, rapidly involuting congenital hemangioma, vascular tumor

## Abstract

This case report presents a rare instance of a giant rapidly involuting congenital hemangioma (RICH) in a neonate. Prenatal ultrasound identified a placenta-like lesion on the right lower limb. Postnatally, a massive, dark purple tumor was observed. Imaging confirmed the diagnosis of RICH with no deep tissue involvement. The lesion underwent complete spontaneous involution within the first year of life without intervention, accompanied by transient thrombocytosis and ulceration during regression. This case demonstrates that even exceptionally large RICH lesions follow the classic, rapid involuting course. It underscores the importance of recognizing characteristic clinical and radiological features to adopt an active observation strategy, thereby avoiding unnecessary invasive treatments. The report highlights the roles of multidisciplinary management and long-term follow-up in ensuring optimal outcomes.

## Introduction

Congenital hemangiomas (CHs) are rare benign vascular tumors that are fully developed at birth, distinguishing them from the more common infantile hemangiomas (IH). Unlike IH, CHs do not undergo postnatal proliferation. Their etiology remains unclear. CHs commonly involve the skin and subcutaneous tissues, with a predilection for the limbs (particularly near joints) and the scalp, and may also affect the liver (1, 2). While many CHs are clinically manageable, large lesions can lead to various life-threatening complications. These include high-output cardiac failure (3), platelet trapping with severe consumptive coagulopathy (Kasabach-Merritt syndrome) (4), profound hypotension potentially leading to brain death, non-immune hydrops (5), and even intrauterine fetal demise (6). Prenatal diagnosis of congenital hemangiomas is challenging, and accurate identification is crucial for optimal prenatal and perinatal management. This case report presents an isolated giant rapidly involuting congenital hemangioma, aiming to illustrate its key characteristics and underscore the importance of its recognition during the initial neonatal assessment.

## Case report

**Figure 1:**
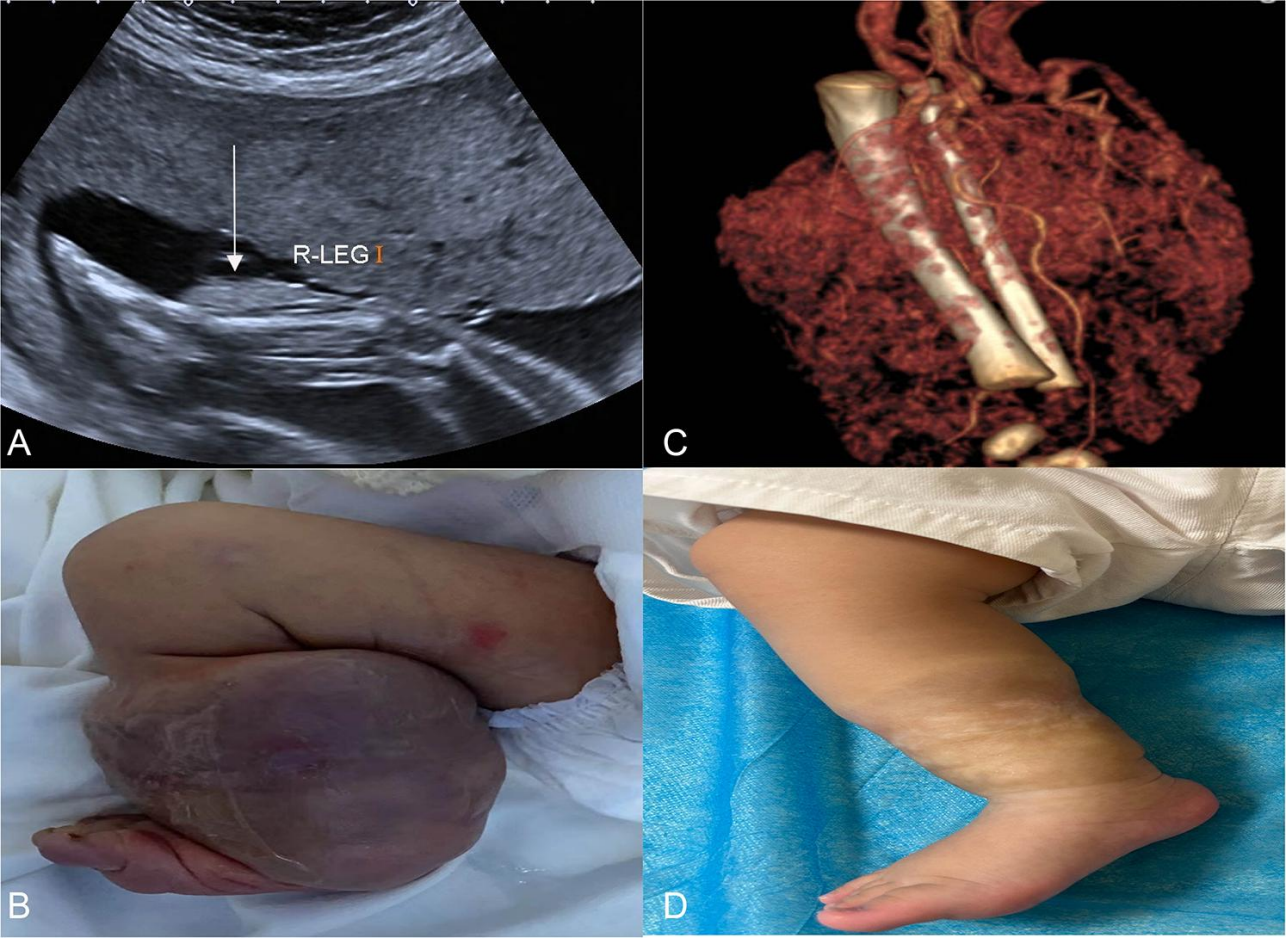
Giant rapidly involuting congenital hemangioma (RICH) of the lower extremity. **(A)** Prenatal ultrasound reveals a mass with a scalloped appearance, exhibiting echogenicity similar to that of the placenta. **(B)** A massive dusky purple mass is observed on the right lower leg, with a circumference of approximately 280 mm, surrounded by a peripheral rim of pallor. **(C)** Contrast-enhanced CT scan reveals tortuous feeding arteries and draining veins within the mass, demonstrating a rapid arterial enhancement with subsequent washout pattern, without involvement of the muscle or bone. **(D)** The mass showed spontaneous regression at the one-year follow-up.

A male neonate was delivered via elective cesarean section at term to a 38-year-old mother, gravida 2 para 1, with an unremarkable pregnancy history. There was no reported exposure to infections such as cold or fever, radiation, or toxic substances during gestation. The family history was negative for congenital malformations or vascular diseases. A routine fetal ultrasound at 18 weeks of gestation revealed no abnormalities. However, a follow-up ultrasound at 23 weeks demonstrated progressive thickening of the skin on the right lower leg. The lesion exhibited a scalloped appearance with a placenta-like echotexture, extending from the knee to the ankle (Figure 1A).

The infant had a body length of 48 cm and a weight of 2900 g. Physical examination revealed a massive, dark purple tumor encircling the right calf, with a circumference of 280 mm. A characteristic peripheral rim of pallor (vasoconstriction halo) was noted surrounding the lesion. Subcutaneous ecchymosis was noted. The mass was fluctuant on palpation, with an irregular texture and ill-defined borders, and did not blanch with pressure (Figure 1B).

Laboratory investigations showed a mildly elevated platelet count of 450 × 10⁹/L. Coagulation studies revealed mild coagulopathy, characterized by slightly prolonged prothrombin time (PT) and activated partial thromboplastin time (APTT), along with mildly elevated D-dimer levels, but with normal fibrinogen levels. These findings did not meet the criteria for the severe consumptive coagulopathy seen in Kasabach-Merritt phenomenon. Echocardiography was performed and ruled out heart failure. Superficial ultrasound demonstrated a heterogeneous, well-circumscribed mass with prominent internal vascularity. Color Doppler examination revealed multiple tortuous feeding arteries with a high-velocity, low-resistance arterial waveform pattern and dilated draining veins, consistent with a high-flow vascular tumor. No arteriovenous shunting was identified. Contrast-enhanced CT was performed due to clinical urgency and the inability to arrange sedated MRI within the required timeframe. In neonates, MRI is generally the preferred imaging modality for vascular anomalies owing to its superior soft-tissue contrast and absence of ionizing radiation (7). In this case, low-dose CT protocol was utilized with weight-adapted contrast dosing to minimize radiation exposure. The CT findings demonstrated a well-circumscribed, heterogeneously enhancing mass with tortuous feeding arteries and draining veins, exhibiting a characteristic rapid arterial enhancement with subsequent washout pattern. The mass was confined to the skin and subcutaneous tissue, confirming no involvement of the underlying muscle or bone (Figure 1C).

At one week of age, the platelet count normalized, and the color of the lesion began to lighten. Within one month, the tumor size significantly decreased, and ulceration developed on its surface. The ulceration was managed with daily wound cleansing using normal saline, application of petrolatum-based ointment under occlusive dressing, and regular assessment for signs of secondary infection. No topical or systemic antibiotics were required, and wound cultures were not obtained as there were no clinical signs of infection. Pain was assessed using the Neonatal Infant Pain Scale (NIPS), and no pharmacological analgesics were needed during the course of ulceration management. The patient was managed conservatively, including wound care for the ulcerated area, infection surveillance, and regular clinical follow-up with comprehensive parental education regarding the expected natural history of RICH. Serial measurements of the lesion were recorded: the circumference decreased from 280 mm at birth to approximately 260 mm at 1 week, 200 mm at 1 month, 150 mm at 3 months, 130 mm at 6 months, and the mass was no longer palpable by 12 months, with the affected limb circumference equivalent to the contralateral side. Follow-up over one year demonstrated spontaneous and complete involution of the lesion (Figure 1D, Table 1). This clinical course is characteristic of a Rapidly Involuting Congenital Hemangioma (RICH). According to a review of the literature, the tumor in this case represents one of the largest RICH lesions reported to date; nevertheless, its natural history followed the classic pattern of rapid involution.

**Table 1:** CARE timeline of clinical course.

Time point	Clinical events
18 weeks gestation	Routine fetal ultrasound: no abnormalities
23 weeks gestation	Follow-up ultrasound: progressive skin thickening on right lower leg; scalloped, placenta-like echotexture from knee to ankle
Birth (Term)	Cesarean delivery; massive dark purple tumor encircling right calf (circumference 280 mm); vasoconstriction halo; subcutaneous ecchymosis; platelet count 450 × 10⁹/L; mild coagulopathy (slightly prolonged PT/APTT, elevated D-dimer); echocardiography normal; ultrasound and CT performed
1 week	Platelet count and coagulation profile normalized; lesion color began to lighten; circumference reduced to approximately 260 mm
1 month	Significant tumor size reduction (circumference approximately 200 mm); surface ulceration developed; wound care initiated
3 months	Continued involution (circumference approximately 150 mm); ulceration healing; no signs of infection
6 months	Marked regression (circumference approximately 130 mm); skin flattening; no residual ulceration
12 months	Complete involution; mild residual skin atrophy and telangiectasia; limb circumference symmetric with contralateral side

## Discussion

According to the updated International Society for the Study of Vascular Anomalies (ISSVA) 2025 classification (8), congenital hemangiomas are categorized into three subtypes: Rapidly Involuting Congenital Hemangiomas (RICHs), Non-Involuting Congenital Hemangiomas (NICHs), and Partially Involuting Congenital Hemangiomas (PICHs) (9).

Rapidly Involuting Congenital Hemangioma (RICH) is a rare vascular tumor typically located on the limbs, head, or neck. Its growth completes *in utero*, and it lacks a postnatal proliferative phase, usually undergoing spontaneous involution within 14 months after birth (10). Management is typically expectant, involving close monitoring of the lesion. A favorable prognosis is indicated if the lesion shows volume reduction within the first year of life (5). This case confirms that RICH can present as a giant, circumferential mass with a placental-like, isoechoic appearance on prenatal ultrasound. The characteristic rapid arterial enhancement with subsequent washout pattern on CT and the transient, self-resolving thrombocytosis observed align with previously described RICH features (11). Although ulceration during the involution phase is uncommon, it generally requires only close monitoring for infection prevention. Conservative observation is appropriate given the benign natural history. The diagnostic, management, and follow-up process of this case provides valuable clinical insights and serves as an important reference for therapeutic decision-making.

The presentation at birth as a giant lower limb mass prompted a broad differential diagnosis, including other congenital vascular tumors (e.g., kaposiform hemangioendothelioma, tufted angioma), vascular malformations (e.g., arteriovenous malformation), and congenital fibrosarcoma (12). Kaposiform hemangioendothelioma (KHE) was considered but deemed unlikely because KHE typically presents with profound thrombocytopenia and Kasabach-Merritt phenomenon (KMP), characterized by consumptive coagulopathy with hypofibrinogenemia and elevated D-dimer, whereas our patient exhibited only mild thrombocytosis (13). Furthermore, KHE tends to be a locally aggressive infiltrative tumor crossing multiple tissue planes, whereas imaging in our case showed a well-circumscribed mass confined to the skin and subcutaneous tissue (14). Arteriovenous malformation (AVM) was excluded based on the absence of arteriovenous shunting on Doppler examination and the lobular architecture of the mass on CT, which is inconsistent with the nidus-type morphology characteristic of AVMs (7). Congenital fibrosarcoma was also considered given the large size and neonatal presentation; however, fibrosarcoma typically presents as a firm, rapidly growing mass with aggressive local behavior and heterogeneous enhancement without the organized vascular lobular pattern seen in our case (11). The definitive diagnosis of RICH relied on the integration of typical clinical presentation, characteristic imaging features, and most crucially, the observed trend of early and rapid involution. Although biopsy was not performed in this case, histopathological examination remains an important diagnostic tool when imaging is inconclusive. RICH characteristically demonstrates GLUT-1 negativity, lobular architecture of capillary-sized vessels, areas of fibrosis, hemosiderin deposition, and thrombosed vessels, distinguishing it from infantile hemangiomas which are GLUT-1 positive (15). This case underscores the importance of recognizing prenatal sonographic features, performing meticulous physical examination at birth, conducting early imaging assessments, and adopting a strategy of “active observation” in non-emergent situations. This approach helps avoid unnecessary invasive interventions for lesions that are destined to regress spontaneously.

To contextualize the size of the present lesion, Table 2 summarizes previously reported large RICH cases from the literature.

**Table 2:** Comparison of large RICH lesions reported in the literature.

Study	Location	Size	Involution	Key features
Berenguer et al. 2003 (16)	Limb/Head	Up to 10 cm	6–14 months	Landmark series; *n* = 13
Braun et al. 2020 (5)	Various	Mean 5 cm	< 14 months	Single-centre; *n* = 25 CH
Qiu et al. 2024 (17)	Various	Mean 6 cm (range 1.5–16 cm)	Mean 10.1 months	Largest prospective study; *n* = 86
Present case	Right calf	Circumference 280 mm	12 months	Circumferential; one of the largest reported

As shown in Table 2, the largest reported RICH diameter in the prospective study by Qiu et al. was 16 cm (17). Our case, with a circumference of 280 mm encircling the entire calf, exceeds this in overall lesion extent, supporting the characterization as one of the largest RICH lesions described to date.

The pathophysiological mechanisms underlying RICH are not fully elucidated but are considered distinct from infantile hemangiomas, potentially involving earlier proliferation of vascular endothelial progenitor cells and premature activation of apoptosis programs (18). The “giant” size of the lesion in this case highlights the necessity of recognizing such entities. Large lesions may lead to local complications such as ulceration, bleeding, mild coagulopathy (with the rare but important consideration of Kasabach–Merritt phenomenon [KMP], a life-threatening consumptive coagulopathy characterized by profound thrombocytopenia and hypofibrinogenemia, typically associated with kaposiform hemangioendothelioma or tufted angioma rather than congenital hemangiomas), or psychosocial concerns due to their conspicuous size. Nonetheless, their rapidly involuting nature dictates a primarily conservative and supportive management approach. The significant regression observed during follow-up in this case reinforces this core characteristic. Notably, this case is remarkable for several reasons: (1) the exceptionally large size of the lesion (circumference of 280 mm, circumferentially encircling the calf), making it one of the largest RICH lesions reported in the literature; (2) the complete spontaneous involution despite its giant size, reinforcing that even very large RICH lesions follow the classic benign natural history; (3) the detailed documentation of the entire evolution from prenatal detection through complete postnatal regression; and (4) the transient thrombocytosis (rather than the more commonly reported thrombocytopenia), contributing to the understanding of hematologic variations in RICH.

Regarding the hematologic findings, the combination of transient thrombocytosis (platelet count 450 × 10⁹/L) and mild coagulopathy is noteworthy. While transient thrombocytopenia and mild coagulopathy have been well-documented in RICH, the presentation of thrombocytosis concurrent with coagulopathy is less commonly reported. The coagulation profile in this case demonstrated mild abnormalities (prolonged PT/APTT and elevated D-dimer) without profound hypofibrinogenemia, safely differentiating it from the life-threatening Kasabach-Merritt phenomenon typically associated with kaposiform hemangioendothelioma. These laboratory parameters normalized spontaneously within the first week. This case highlights the importance of performing comprehensive coagulation screening at diagnosis for giant RICH to properly characterize the hematologic profile and rule out severe consumptive coagulopathy.

There is no standardized treatment protocol for RICH. The cornerstone of management involves: 1) Accurate Diagnosis: Utilizing clinical and imaging modalities to exclude other conditions requiring urgent intervention; 2) Complication Prevention and Management: Providing appropriate local care for ulceration, pain, or infection; and 3) Expectant Observation: Thoroughly educating parents about the natural history and establishing a regular follow-up schedule to monitor involution, as well as to assess limb development and function. The successful management of this case relied on a multidisciplinary team (involving neonatology, dermatology, and radiology) and comprehensive family education.

Both the literature and the present case indicate that after complete involution of a RICH, the skin appearance is generally favorable, though sequelae such as telangiectasias, skin atrophy, hypopigmentation, or redundant skin may persist. Post-involution, attention must be paid to potential discrepancies in limb length or girth. Therefore, long-term follow-up should extend beyond the lesion itself to include monitoring of limb symmetry, gait, and overall function. Unfortunately, the original spectral Doppler ultrasound images were not archived at sufficient resolution for publication; this represents a limitation of the present report. The infant in this report will continue to undergo periodic follow-up to monitor for possible long-term sequelae and to facilitate timely intervention if necessary.

## Conclusion

This report describes a case of RICH with characteristic imaging findings, demonstrating its evolution and complete regression within the first year of life. Recognizing its characteristic clinical and radiological presentation helps prevent overtreatment, shifting the management focus toward active observation, complication management, thorough patient family education, and long-term follow-up.

## Data Availability

The original contributions presented in the study are included in the article/Supplementary Material, further inquiries can be directed to the corresponding author.
